# Physiological Role of Glutamate Dehydrogenase in Cancer Cells

**DOI:** 10.3389/fonc.2020.00429

**Published:** 2020-04-09

**Authors:** Rafael Moreno-Sánchez, Álvaro Marín-Hernández, Juan C. Gallardo-Pérez, Silvia C. Pacheco-Velázquez, Diana X. Robledo-Cadena, Joaquín Alberto Padilla-Flores, Emma Saavedra, Sara Rodríguez-Enríquez

**Affiliations:** Departamento de Bioquímica, Instituto Nacional de Cardiología, Ciudad de México, Mexico

**Keywords:** ammonium, metastatic cancer cells, GDH kinetics, cooperativity, monod–wyman–changeux model

## Abstract

${\text{NH}}_{4}^{+}$ increased growth rates and final densities of several human metastatic cancer cells. To assess whether glutamate dehydrogenase (GDH) in cancer cells may catalyze the reverse reaction of ${\text{NH}}_{4}^{+}$ fixation, its covalent regulation and kinetic parameters were determined under near-physiological conditions. Increased total protein and phosphorylation were attained in ${\text{NH}}_{4}^{+}$-supplemented metastatic cells, but total cell GDH activity was unchanged. Higher *V*_max_ values for the GDH reverse reaction vs. forward reaction in both isolated hepatoma (HepM) and liver mitochondria [rat liver mitochondria (RLM)] favored an ${\text{NH}}_{4}^{+}$-fixing role. GDH sigmoidal kinetics with ${\text{NH}}_{4}^{+}$, ADP, and leucine fitted to Hill equation showed *n*_*H*_ values of 2 to 3. However, the *K*_0.5_ values for ${\text{NH}}_{4}^{+}$ were over 20 mM, questioning the physiological relevance of the GDH reverse reaction, because intracellular ${\text{NH}}_{4}^{+}$ in tumors is 1 to 5 mM. In contrast, data fitting to the Monod–Wyman–Changeux (MWC) model revealed lower *K*_m_ values for ${\text{NH}}_{4}^{+}$, of 6 to 12 mM. *In silico* analysis made with MWC equation, and using physiological concentrations of substrates and modulators, predicted GDH N-fixing activity in cancer cells. Therefore, together with its thermodynamic feasibility, GDH may reach rates for its reverse, ${\text{NH}}_{4}^{+}$-fixing reaction that are compatible with an anabolic role for supporting growth of cancer cells.

## Introduction

It has been proposed that the primary metabolic function of glutamate dehydrogenase [GDH; Glu + NAD(P)^+^ + H_2_O **<->** 2-OG + NH_3_ (or ${\text{NH}}_{4}^{+}$) + NAD(P)H + H^+^; EC: 1.4.1.3] is to produce ammonia, or the toxic ion ammonium (${\text{NH}}_{4}^{+}$), either for urea synthesis in liver, or for direct clearance in kidney (1–5), or to produce 2-oxoglutarate (2-OG) for export to neurons from astrocytes (6). In the forward, oxidative deaminating reaction, GDH provides reducing equivalents as NADPH for oxidative stress management in the mitochondrial matrix and 2-OG for Krebs cycle and oxidative phosphorylation. This canonical role of GDH is a consequence of the efficient transference by transaminases of the α-amino group of several amino acids to 2-OG forming glutamate (Glu). However, it has been shown that the predominant source of ammonium in liver derives from glutamine, not from glutamate (6), and the GDH reaction seems positioned near its thermodynamic equilibrium (3). Moreover, significant GDH activity is also present in mitochondria of nonureogenic organs, such as heart, skeletal muscle, and brain, and the gene encoding GDH1 in humans is expressed in all tissues, whereas the GDH2 gene expression is specific for nerve tissues and testis (4, 5), although the kinetic properties of both GDH isoenzymes are not significantly different (4). Hence, an ammonium-producing role for GDH can be contested. It appears that the mammalian homohexameric GDH may also have other functions different from that of ammonium supplier, for instance, that of nitrogen assimilation, as well as accessory roles, such as its binding to chromosome X-linked inhibitor of apoptosis protein (XIAP) preventing XIAP inhibition on caspases and thereby promoting cell death (7) and its histone H3-specific tail proteolytic activity in the nucleus (8).

A nitrogen-storing (aminating) role for liver GDH was early proposed by McGivan and Chappell (9) based on their analysis of the rates of the enzyme and surrounding pathways and GDH reaction equilibrium constant. Furthermore, the equilibrium constant (*K*_EQ_ = [2-OG] × [NADPH] × [H^+^] × [${\text{NH}}_{4}^{+}$]/[Glu] × [NADP^+^]) of the GDH reaction, of ~1 × 10^−15^ M^2^ (10) that becomes ~1 × 10^−8^ M at pH 7.0, indicates that the reverse reaction (i.e., glutamate formation from 2-OG) is thermodynamically favorable under physiological conditions, when at least low micromolar ammonium concentrations are present. On this issue, it is worth recalling that the directionality of a given reaction is dictated only by its *K*_EQ_ value and the actual mass action ratio of [products]/[substrates], even within pathways working in steady state, in which the reaction is kept away from equilibrium because the coupling, adjacent reactions do not allow the products to accumulate. What the kinetic properties of a given enzyme govern, either whether it is down-regulated, overexpressed, or mutated, is the rate at which the reaction proceeds in the forward or reverse direction.

In addition to lactate, ammonium can also be found in the solid tumor microenvironment at levels significantly greater (0.14–5 mM) than those (0.027–0.05 mM) of the healthy organ microenvironments and plasma (5, 11–14). This is caused by the tumor accelerated glutamine metabolism (15–17), as well as by ammonium simple diffusion across the plasma membrane and tumor defective vasculature. High ammonium concentrations are extremely toxic for normal cells and organs, being particularly critical under metabolic acidosis. In the brain, ammonium primarily affects neurons, because it competes with K^+^ for inward transport via Na^+^/K^+^ ATPase and Na^+^K^+^ Cl^−^ cotransporter (18), and hence ion homeostasis, electric resting and action potentials, and nerve transmission are compromised. In contrast, high ammonium seems innocuous for human cancer cells and rather promotes partial restoration of proliferation of glutamine-depleted cancer cells (19–21) and increased rates of proliferation and tumor growth of glutamine-supplemented cancer cells (5, 21).

Increased transcription of the GDH genes is found in many cancer types (5, 22–24). Although transcription of glutamine synthetase (another enzyme involved in ammonium assimilation) is also increased in some cancer cells (5, 25), it was recently shown by metabolic tracing analysis with [^15^N]amide-glutamine or [^15^N]-NH_4_Cl in breast and prostate cancer cells and tumor xenografts in mice that ammonium was primarily assimilated to glutamate through the GDH reverse reaction and then to proline, glutathione, and direct products of the glutamate-dependent transaminase reactions (5, 26); no urea cycle intermediates were labeled, discarding a role for carbamoyl phosphate synthetase I (the mitochondrial matrix isoform) in cancer ammonium assimilation. Furthermore, kinetic modeling of the mitochondrial NADPH/GSH/ROS pathway predicted that, at physiological values of the NADPH/NADP^+^ (of 0.5–2) and 2OG/Glut (of 0.01–0.1) ratios, GDH behaved as an NADPH consumer catalyzing its reverse reaction, which becomes thermodynamically favored by the presence of micromolar concentrations of ammonium (27). Theoretical modeling of central carbon and nitrogen metabolism also predicted that, when cells take up external ammonium, GDH reverse reaction is required for supporting cell proliferation (28). Indeed, addition of millimolar ammonium to the culture medium significantly increases the growth of human breast MCF-7 and T47D cancer cells (5, 21).

However, a direct and essential role of GDH in ammonium assimilation of cancer cells appears controversial because the GDH activities (*V*_max_) are lower in cancer mitochondria, and GDH shows very low affinity for ammonium, with apparent Michaelis–Menten constants (*K*_m_ or *K*_0.5_) of 8 to 80 mM (27, 29–35). It is noted that these kinetic parameters have been calculated from experimental data fitted to the Hill equation for sigmoidal kinetic behavior and under variable and non-saturating ADP concentrations, an allosteric activator; in addition, the assay pH values used have not been within the physiological range.

A systematic analysis of the GDH activity in cancer mitochondria has not been yet undertaken. Therefore, in the present study, the GDH kinetics was also examined in both liver and hepatoma mitochondria. The simple Hill equation and the more complex Monod–Wyman–Changeux (MWC) equation for exclusive binding (36) were tested as models to fully describe the sigmoidal and cooperative kinetic behavior of GDH. The latter model was able to determine GDH *V*_max_ values, *K*_s_ or *K*_m_ for substrates, catalytic efficiencies (*V*_max_/*K*_m_), activation constants (*K*_a_) for ADP and leucine, inhibition constant (*K*_i_) for GTP, and other relevant parameters related to its cooperative behavior. These GDH kinetic properties, together with the determination of the GDH reaction metabolites in the cell, provided the required information to envision the mechanisms by which GDH may play a key role, as an inorganic nitrogen-fixation device in cancer cells, for amino acids and nucleotides syntheses and cell growth.

## Results

### Ammonium Stimulates Growth of Human Metastatic Cancer Cells

**Figure 1:**
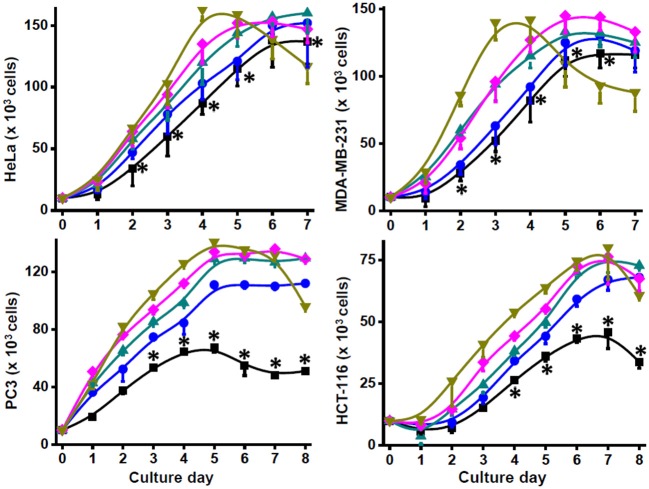
Growth of human metastatic cancer cells with ammonium. Cancer HeLa, MDA-MB-231, PC3, and HCT116 cells (20 × 10^3^ cells/well) were grown in 96-well plates in the presence of 0 (■), 0.5 (•), 1 (▴), 5 (♦), and 10 (▾) mM NH_4_Cl. Cell growth was monitored by counting cellular density every 24 h. Viability was >90% in all culture conditions. Data shown represent the mean ± SD of at least three different preparations. Statistical analysis was performed using one-way ANOVA with Scheffé comparison test. *P < 0.01 vs. 1, 5, or 10 mM NH_4_Cl.

Addition of NH_4_Cl (0.1–10 mM) to bidimensional (2-D) human HeLa, MDA-MB-231, PC3, HTC116, and Colo205 metastatic cancer cell cultures clearly decreased their duplication times, stimulated their proliferation rates, and allowed to reach higher final cell densities at the stationary phase (Figure 1; Table 1). In contrast, growth rates and final cell densities of 2-D human breast cancer MCF-7, cervix SiHa, prostate DU145, and lung A549 cancer cells, which have low metastatic potential, were not affected by ammonium supplementation of 0.5 to 10 mM NH_4_Cl, except for a significant stimulatory effect on μ, the specific cell growth rate, and maximal density at 10 mM in MCF-7 cells (Table 1), and increased proliferation rates at 0.1 and 0.5 mM NH_4_Cl in DU145 cells (data not shown) but significantly decreased cell densities at ammonium concentrations higher than 1 mM (Table 1).

**Table 1:** Effect of ammonium supplementation on cancer cell proliferation.

	**Duplication time (h)**			**μ** **(h**^**-1**^**)**			**Maximal cellular densities (×10**^**3**^**)**		
	**Control**	**NH**_**4**_**Cl (mM)**		**Control**	**NH**_****4****_**Cl (mM)**		**Control**	**NH**_**4**_**Cl (mM)**	
		**+5**	**+10**		**+5**	**+10**		**+5**	**+10**
**Metastatic cells**
MDA-MB-231	32 ± 1	26 ± 3 ^*^	25 ± 1^*^	0.47 ± 0.05	0.61 ± 0.06^*^	0.9 ± 0.07^*^	82 ± 16	145 ± 13^*^	142 + 17^*^
HeLa	31 ± 3	25.5 ± 2^*^	24 ± 2^*^	0.63 ± 0.04	0.77 ± 0.03^*^	0.8 ± 0.05^*^	87 ± 9	153 ± 13^*^	163 ± 9^*^
PC3	36 ± 2	28 + 2^*^	26 ± 1^*^	0.57 ± 0.06	1.01 ± 0.04^*^	1.05 ± 0.03^*^	68 ± 3.5	136 ± 2^*^	141 ± 1.4^*^
HCT116	69 ± 3	45 ± 1^*^	39 ± 2^*^	0.36 ± 0.05	0.49 ± 0.03^*^	0.51 ± 0.02^*^	45 ± 7	76 ± 11^*^	80 ± 13^*^
Colo 205	80 ± 5	48 ± 2^*^	44 ± 4^*^	0.53 ± 0.02	0.62 ± 0.05^*^	0.63 ± 0.04^*^	45 ± 7	68 ± 6^*^	66 ± 6^*^
**Low metastatic cells**
MCF-7	34 ± 1	37 ± 2	45 ± 6	0.34 ± 0.05	0.38 ± 0.02	0.69 ± 0.03^*^	44 ± 7	72 ± 6^*^	76 ± 5^*^
SiHa	48 ± 3	51 ± 4	55 ± 5	0.60 ± 0.04	0.58 ± 0.05	0.55 ± 0.07	90 ± 6	86 ± 7	82 ± 5
DU145	26 ± 2	32 ± 2^*^	35 ± 3^*^	1.0 ± 0.06	0.47 ± 0.07^*^	0.55 ± 0.04^*^	106 ± 8	92 ± 6	85 ± 4^*^
A549	25 ± 3	20 ± 2	17 ± 3^*^	0.78 ± 0.05	0.82 ± 0.04	0.85 ± 0.06^*^	41 ± 5	49 ± 4	56 ± 2^*^
**Non-cancer cells**
3T3	26 ± 0.5	46 ± 2^*^	60 ± 1^*^	0.7 ± 0.02	0.43 ± 0.1^*^	0.32 ± 0.003^*^	131 ± 5	45 ± 3^*^	36 ± 2^*^
HFF-1	32 ± 7	38 ± 12	55 ± 2^*^	0.75 ± 0.1	0.65 ± 0.08	0.4 ± 0.1^*^	93 ± 5	60 ± 10^*^	46 ± 3^*^

*The number of independent cell cultures assayed (n) was 3. Statistical analysis was performed using one-way ANOVA with Scheffé comparison test*.

* *P ≤ 0.05 or 0.01 vs. control; the cell viability in all conditions was >90%. The maximal cellular densities were reached for HeLa and MDA-MB-231 cells at day 4; for PC3, 3T3, HFF-1, and DU145 cells at day 5; for Colo-205, MCF-7, A549, and SiHa cells at day 6; and for HCT 116 cells at day 7*.

Ammonium toxicity was not apparent (cell morphology was preserved, and viability was higher than 90%), except for a moderate decrease in cell density at 10 mM in the stationary phase of 2-D cultures of HeLa, MDA-MB-231, PC3, and HTC116 cells (Figure 1). In contrast, ammonium severely decreased growth of non-cancer mouse 3T3 fibroblasts and human HFF-1 fibroblasts with IC_50_ values around 1 mM (data not shown); higher ammonium concentrations significantly affected duplication times, specific growth rates, and maximal cell densities of mouse and human fibroblasts (Table 1). Glutamine removal from the culture medium induced an acute decrease in the growth rates of HeLa and MDA-MB-231 cells; NH_4_Cl (1–10 mM) addition did not rescue their growth (data not shown). In this last regard, it is noted that cell culture in glutamine-lacking medium is not a physiologically realistic condition. In addition, transcription of genes involved in proliferation and other processes in cancer cells may be regulated by glutamine (5, 19, 37). Therefore, ammonium supplementation experiments in glutamine-depleted media were not further pursued.

**Figure 2:**
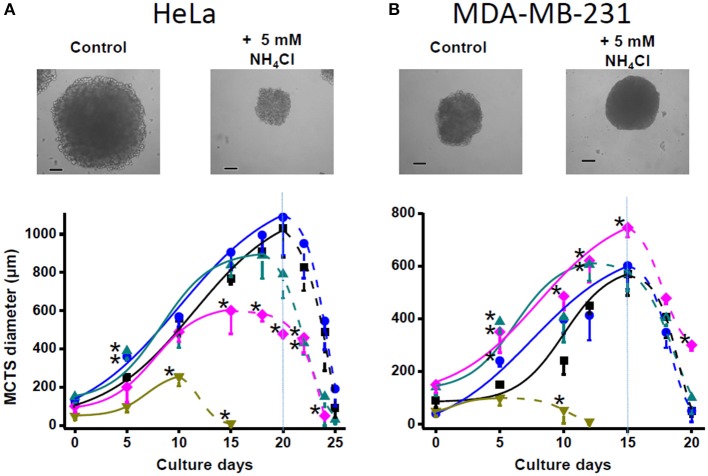
Effect of ammonium supplementation on MCTS growth. **(A)** HeLa and **(B)** MDA-MB-231 cells (2 × 10^4^ cells/mL) were seeded in 2% (wt/vol) agarose-coated culture dishes in DMEM with 0 (■), 0.5 (•), 1 (▴), 5 (♦), and 10 (▾) mM NH_4_Cl. Spheroid micrographs were taken at day 20 (HeLa) or day 15 (MDA-MB-231); bars represent 200 μm. Fresh DMEM medium was replaced every 3 days. The spheroid growth was determined at the indicated times by measuring MCTS diameter. Data shown represent the mean ± SD of at least 30 MCTS (10 MCTS from each preparation), *n* = 3 different preparations. Statistical analysis was performed using one-way ANOVA with Scheffé comparison test. *P < 0.01 vs. non-treated MCTS.

Ammonium supplementation to the tridimensional multicellular tumor spheroids of HeLa cells did not stimulate growth, but in fact 5 and 10 mM ammonium inhibited it (Figure 2A). For MDA-MB-231 multicellular tumor spheroid (MCTS), which were significantly smaller than those of HeLa MCTS, ammonium in the 0.5–5 mM range promoted enhanced growth rates, whereas it was clearly toxic at 10 mM (Figure 2B).

### Effect of Ammonium Supplementation on GDH Protein Level and Activity

**Figure 3:**
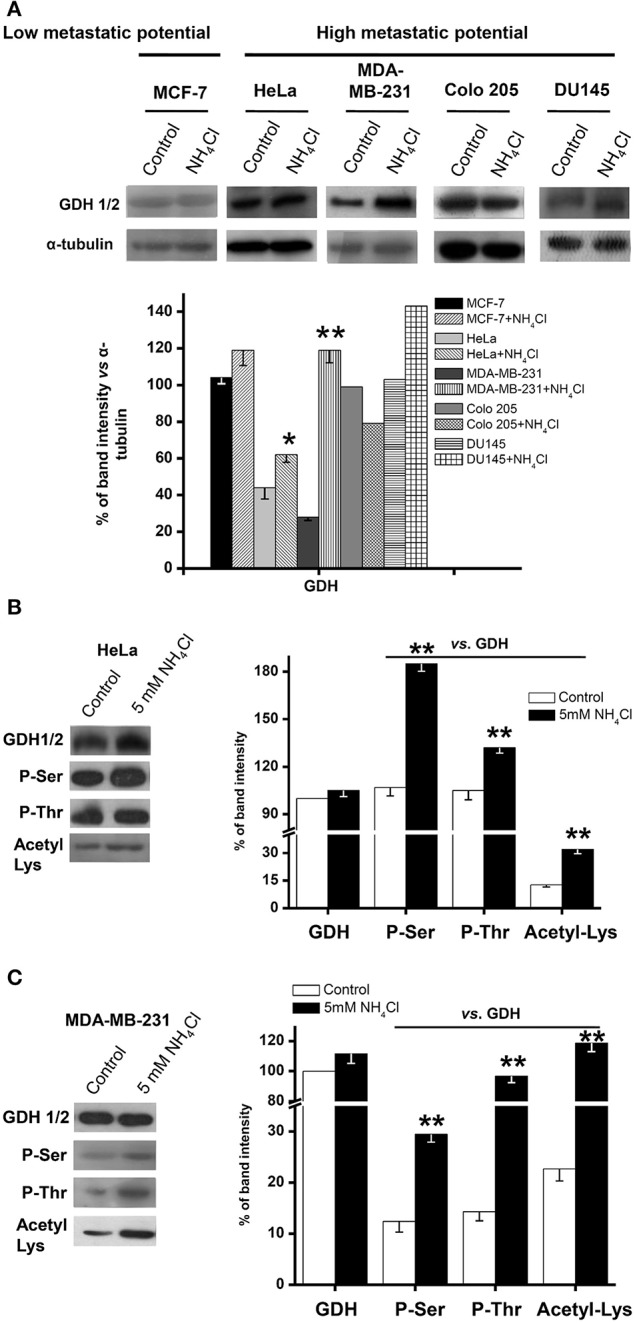
Glutamate dehydrogenase covalent modifications in human metastatic cancer cells. Total GDH protein content by Western blotting **(A)** and GDH phosphorylation and acetylation status by immunoprecipitation **(B,C)** were determined, as described in *Materials and Methods*, in MCF-7, HeLa, MDA-MB-231, Colo 205, and DU145 cells exposed to 5 mM NH_4_Cl (except for DU145, which was exposed to 1 mM) for 5 days in 2-D cultures. Representative results are shown. α-Tubulin was used as loading control and for data normalization in the Western blot experiments **(A)**; the control GDH protein signal of each individual experiment (no ${\text{NH}}_{4}^{+}$ added) was used for initial normalization of the GDH signal with ${\text{NH}}_{4}^{+}$ in the immunoprecipitation assays **(B,C)**, followed by comparison of the phosphorylation and acetylation signals against their respective total immunoprecipitated GDH (control or 5 mM NH_4_Cl) signal. Densitometric analysis represents the mean ± SD of three independent cell cultures (except for Colo 205 and DU145, *n* = 2). Statistical analysis was performed using one-way ANOVA with Scheffé comparison test. *P < 0.05, **P < 0.01 vs. control.

Ammonium supplementation induced either a small (HeLa, DU145) or large (MDA-MB-231) increase, or no change (Colo 205), in the total GDH (GDH1 + GDH2) protein content in metastatic cancer cells, and no change in non-metastatic (MCF-7) cells (Figure 3A).

The total GDH reverse reaction activity (GDH1 + GDH2) with NADPH as cosubstrate in HeLa and MCF-7 cells was detectable and significant (35–70 mU/mg protein), and also similar to that determined in AS-30D hepatoma cells. However, these GDH activities did not allow for reliable measurements at variable ammonium for determining *K*_m_ values; significant interference of cell suspensions due to turbidity, despite the addition of triton x-100, occurred on the determination of GDH activity. Addition of 5 mM NH_4_Cl to the culture medium and growth for 5 days did not alter the total GDH activity or induced a slight (~20%) decrease (data not shown) in HeLa and MCF-7 cells.

### Covalent GDH Regulation

Glutamate dehydrogenase may undergo a variety of posttranslational modifications, which apparently may also affect activity (38–40). Indeed, immunoprecipitation assays revealed that GDH in HeLa and MDA-MB-231 cells showed significant phosphorylation and acetylation, which were further increased by 5 day growth in the presence of ammonium (Figures 3B,C).

**Figure 4:**
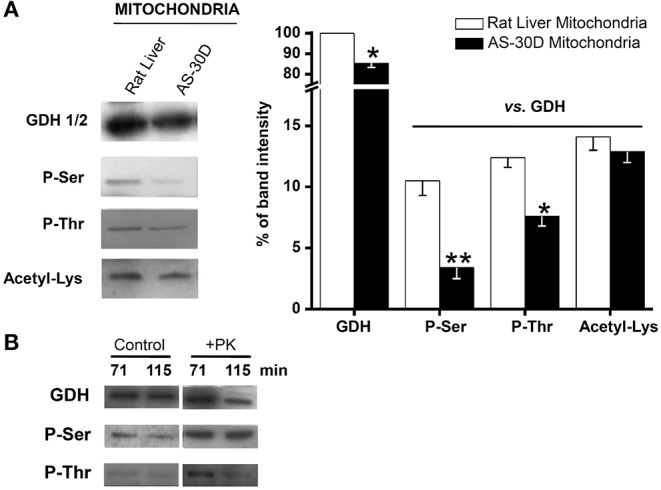
Phosphorylation and acetylation status of GDH in isolated mitochondria. Representative Western blots and relative protein contents are shown. Data represent the mean ± SD of three different preparations. **(A)** The RLM GDH protein signal was used for initial normalization of the HepM GDH signal, followed by comparison of the phosphorylation and acetylation signals against their respective GDH (control or 5 mM NH_4_Cl) signal. **(B)** Phosphorylation of HepM GDH by commercial phosphorylase kinase. HepM fractions (10 mg protein/mL) were incubated at 30°C for the indicated times with 41 mM α-glycerophosphate, 20 mM Tris pH 7.4, 1 mM CaCl_2_, 5 mM ATP, 7 mM MgCl_2_, 300 mM trehalose, and 40 U rabbit muscle phosphorylase kinase (PK). Controls were also carried out under the same conditions, but PK was omitted from the mix reaction. Statistical analysis was performed using one-way ANOVA with Scheffé comparison test.*P < 0.05, **P < 0.01 vs. RLM.

As a direct control and to discard the participation of the nuclear GDH2 isoform, mitochondria isolated from AS-30D hepatoma ascites cells and rat liver were also used to assess mitochondrial matrix GDH1 covalent modifications. The GDH1 content in AS-30D hepatoma mitochondria (HepM) was slightly lower than that of RLM. Furthermore, phosphorylation of Ser and Thr residues was also significantly lower in HepM GDH1, whereas Lys acetylation was similar to that of RLM GDH1 (Figure 4A). Ser phosphorylation was the covalent modification that better correlated with GDH1 activity, because it was approximately three times lower in HepM vs. RLM (Figure 4A), which was similar to the difference in activity (see below). HepM GDH1 phosphorylation (Figure 4B), by a commercial rabbit muscle phosphorylase kinase, produced a moderate but significant increase in activity of 45% ± 39% (*n* = 4).

### GDH Reverse (Aminating) Activity in Hepatoma Mitochondria

The apparent affinity of GDH1 for ammonium is low (the reported *K*_0.5_ values for ammonium are in the 15–60 mM range), which raises doubts on the physiological significance of the presumed GDH1 N-fixing role because ammonium physiological concentrations are much lower. To solidly establish whether GDH1 is able to catalyze its reverse reaction under physiological conditions in cancer cells, a systematic analysis of its kinetic properties is required, in which the affinity constant for ammonium is adequately determined.

The most common GDH1 assay reaction medium usually contains EDTA, a divalent metal cation chelating agent, because it is known that Mg^2+^ may inhibit its activity. However, in the 0- to 0.4-mM range of added MgCl_2_, GDH1 activity was not affected; at 1 mM Mg^2+^, <10% inhibition was attained, and at 2 mM Mg^2+^, ~20% inhibition was achieved. Null Mg^2+^ effects on GDH1 activity have also been previously reported (31). These observations suggested that the regulatory roles of ADP and GTP on GDH are independent on whether the nucleotides are bound to the enzyme as Mg complexes or free forms. Spermidine has been also claimed to inhibit GDH, but in our hands, this polyamine in the 0–5 mM range was innocuous and at 20 mM indeed inhibited GDH activity by 20% (data not shown). GDH1 exhibited a marked hysteresis after several minutes of reaction, depending on the incubation conditions; however, this behavior was not further explored. Moreover, for an appropriate kinetic analysis, initial rate determinations were used because only these can be reliably associated to the added substrate concentrations (before they start changing), and products have not been accumulated to significant levels that may affect enzyme rate.

**Figure 5:**
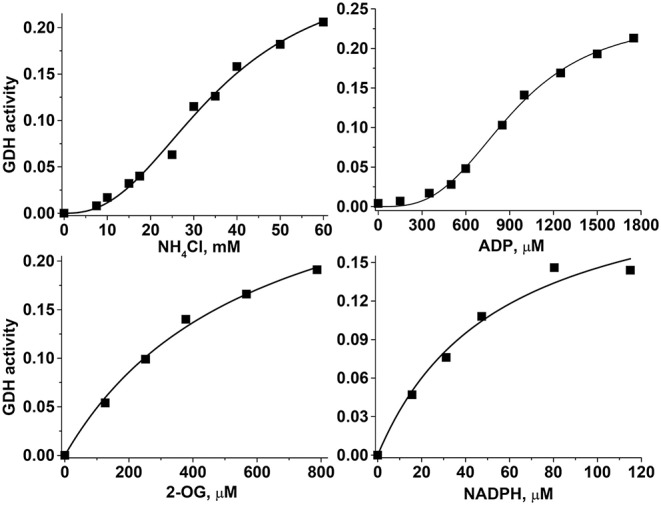
Kinetics of the hepatoma mitochondria reverse GDH reaction. For these representative experiments, 219 μg protein of HepM was incubated in KME buffer at pH 7.20 and 37°C. The saturation curves with variables NH_4_Cl and ADP were fitted to the Hill equation, whereas the saturation curves with variables 2-OG and NADPH were fitted to the Michaelis–Menten equation. The units of the GDH activity were Δabsorbance at 340 nm min^−1^.

The sigmoidal behavior regarding ${\text{NH}}_{4}^{+}$ and ADP and the hyperbolic behavior regarding 2-OG and NADPH of the HepM GDH1 activity (Figure 5) were highly similar to that displayed by RLM GDH (data not shown). The sigmoidal patterns were fitted to the Hill equation that yields *K*_0.5_ values for the variable substrate, and which are approximated but not proper *K*_m_ or *K*_s_ (*k*_−1_/*k*_1_, rapid equilibrium constants) values. The kinetic analysis was carried out at the indicated pH values to encompass the mitochondrial matrix physiological pH range of 7.2 to 8.2 (41, 42); more alkaline pH values are reached only in the absence of Pi, which is not a physiological condition. The only marked effect of higher pH was an increased *K*_m_ for NADPH and lower *K*_0.5_ for ADP in both mitochondrial types (Table 2). Indeed, the pH profile of the GDH1 reverse activity in both mitochondrial types showed maximal rates in the 7.0–7.5 range, sharply decreasing at lower and higher pH values (data not shown). A similar pH profile was reported for the ox liver GDH forward reaction (43), as well as the human GDH1 reverse reaction (34).

**Table 2:** Kinetic parameters of mitochondrial GDH for the reverse reaction derived from the Hill equation.

**GDH kinetic parameters**	**pH 7.2**		**pH 7.5**	
	**HepM**	**RLM**	**HepM**	**RLM**
*V*_max_, mU/mg protein	202 ± 63 (9)^*^	818 ± 168 (10)	168 ± 39 (6)^*^	725 ± 179 (5)
*K*_0.5_ ${\text{NH}}_{4}^{+}$, mM *n_*H*_*	25.2 ± 8 (9) 2.12 ± 0.7 (9)	23.9 ± 3.7 (9) 1.97 ± 0.8 (9)	26.8 ± 7.2 (6) 1.5 ± 0.7 (6)	18.2 ± 5.9 (5) 1.4 ± 0.4 (5)
*V*_max_/*K*_0.5_ ${\text{NH}}_{4}^{+}$, min^−1^ mg^−1^ mL^−1^	0.008	0.034	0.006	0.039
*K*_0.5_ ADP, μM *n_*H*_*	564 ± 167 (4) 2.77 ± 0.7 (4)	567 ± 125 (3) 2.4 ± 0.3 (3)	281 ± 134 (4) 2.6 ± 0.4 (4)	285 (2) 2.34 (2)
*K*_0.5_ Leu, mM *n_*H*_*	2.6 (2) 2.8 (2)	3.9 (2) 2.75 (2)	ND	ND
*K*_m_ 2-OG, μM	442 ± 160 (5)	371 ± 232 (6)	733 ± 325 (4)	396 ± 75 (3)
*V*_max_/*K*_m_ 2-OG, min^−1^ mg^−1^ mL^−1^	0.45	2.20	0.22	1.83
*K*_m_ NADPH, μM	46 ± 14 (6)	56.6 ± 26 (3)	109 ± 4 (3)	133 ± 28 (3)
IC_50_ GTP, μM	114 ± 29 (4)	139 ± 21 (4)	147 ± 32 (3)	244 (2)

*N.D., not determined; Leu, leucine. Statistical analysis was performed using Student t-test*.

* *P < 0.01 vs. RLM. The number of independent preparations assayed is indicated between parentheses*.

The *V*_max_ and catalytic efficiency (*V*_max_/*K*_m_) values were markedly (4- to 8-fold) lower in HepM (Table 2). However, the ligand binding parameters were similar between HepM and RLM, except for a slightly higher IC_50_ value for GTP at pH 7.5 in HepM GDH1. The Hill coefficient values lower than 3 suggested that GDH has a moderate cooperativity among its six subunits. Similar Hill coefficient values have been previously reported (32, 35).

The *K*_0.5_ and Hill coefficient values determined were within the range of values (*K*_0.5NH4+_ = 6.5–80 mM, *K*_m2OG_ = 0.47–4.5 mM, *K*_mNADPH_ = 0.02–0.12 mM, and *n*_*H*_= 1.6–2.4) reported for recombinant human GDH isoforms (GDH1 and GDH2), bovine liver GDH, Ox brain GDH, dogfish liver GDH, rat brain GDH, and bovine brain GDH isoforms, at the pH range of 7.4–8.7 (29, 33–35, 44, 45). It should be noted that the lower *K*_0.5_ values for ammonium were attained at alkaline pH values (33–35, 44).

### GDH1 Reverse (Aminating) Activity Can Be Fitted to the MWC Model

**Figure 6:**
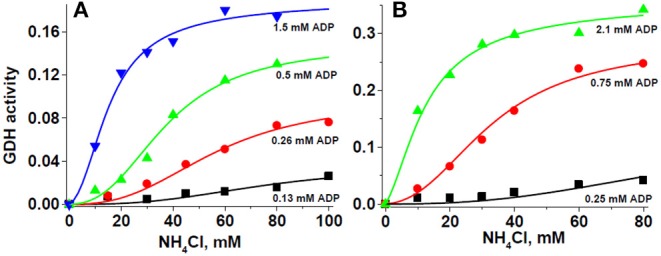
Glutamate dehydrogenase kinetics obeys the ligand exclusive binding MWC model. For these representative experiments, 280 μg protein of HepM **(A)**, and 90 μg protein of RLM **(B)** were incubated in KME + HEPES buffer at pH 7.50 and 37°C. The solid lines represent the simultaneous global fitting of all experimental points to the exclusive ligand binding MWC Equation 1. The χ2 values of the non-linear regression analyses were 0.00003 and 0.00013 for HepM GDH and RLM GDH, respectively. The *V*_max_ values in ΔAbs/min at each ADP concentration were 0.037 ± 0.009 (0.13 mM ADP), 0.099 ± 0.010 (0.26 mM ADP), 0.153 ± 0.008 (0.5 mM ADP), and 0.193 ± 0.011 (1.5 mM ADP) for HepM GDH; and 0.131 ± 0.113 (0.25 mM ADP), 0.295 ± 0.022 (0.75 mM ADP), and 0.367 ± 0.024 (2.1 mM ADP) for RLM GDH.

The sigmoidal kinetic behavior of GDH1 regarding the substrate ${\text{NH}}_{4}^{+}$ and the activators ADP and leucine has been commonly fitted to the Hill equation, $v=\frac{Vmax{\left[S\right]}^{n_{H}}}{K^{\prime }+{\left[S\right]}^{n_{H}}}$. In this equation, cooperativity is assessed by the Hill coefficient *n*_*H*_ value, whereas *K'* does not represent directly a measurement of affinity, although some researchers have interpreted as such. The Hill equation does not allow for estimation of affinities for allosteric activators and inhibitors either. Therefore, an effort was made to fit the experimental GDH1 data to the MWC equation for ligand exclusive binding (36). Thus, Equation 1 (see below) does allow the experimental determination of actual ligand affinity values. Initial attempts yielded poor fitting because the number of interacting subunits (*n*) was fixed to 6, and *V*_max_ was considered to be unique. However, analysis of Figure 5 and other similar results indicated that ADP was an essential allosteric activator because no significant activity was displayed in its absence, and the apparent *V*_max_ clearly changed, depending on the ADP concentration added. Then, when it was assumed that *V*_max_ changes with the ADP concentration (*V*_max_,_ADP_), and *n* was allowed to freely vary, the data fitted MWC Equation 1 exceptionally well (Figure 6).

MWC equation:

(1) $$\begin{array}{l} v=\frac{Vmax_{ADP}\frac{\left[NH_{4}^{+}\right]}{Km_{NH4+}}{\left(1+\frac{\left[NH_{4}^{+}\right]}{Km_{NH4+}}\right)}^{n-1}}{L^{\prime }+{\left(1+\frac{\left[NH_{4}^{+}\right]}{Km_{NH4+}}\right)}^{n}} \end{array}$$

Modification of *L* by allosteric inhibitor:

$$\begin{array}{l} L^{\prime }=L{\left(1+\frac{\left[GTP\right]}{Ki_{GTP}}\right)}^{n} \end{array}$$

Modification of *L* by allosteric activator

$$\begin{array}{l} L^{\prime }=\frac{L}{{\left(1+\frac{\left[ADP\right]}{Ka_{ADP}}\right)}^{n}} \end{array}$$

Surprisingly, the *K*_m_ values for ammonium were significantly lower, at approximately 9 mM at pH 7.5 (Table 3), than the *K*_0.5_ values derived from the Hill equation (Table 2). The *K*_a_ values for ADP were in the submillimolar range, well within the ADP physiological concentrations. It is noted that this is the first time that affinity (1/*K*_m_; 1/*K*_a_) values are produced for the cooperativity ligands of GDH1. Furthermore, *n* was not near 6, the actual number of GDH1 subunits, but rather it was near 3 (Table 3). Linearization of the MWC equation for exclusive ligand binding by using the Horn–Bornig equation (36) rendered *n* values also close to 3 (data not shown). In turn, the large *L* values indicate that GDH1, in the absence of its essential activator ADP, is preferentially stabilized as an inactive form. Ammonium at saturating concentrations (>50 mM) was unable to trigger cooperativity and activity in the absence of ADP; in other words, catalysis was negligible with no ADP. With ADP, the T inactive conformation transforms into an active R state. The exclusive binding MWC model with variable (ADP-dependent) *V*_maxADP_ (Equation 1) also simulated that the *V*_max_ and catalytic efficiency (*V*_max_/*K*_m_) values (estimated at saturating ADP concentrations) of GDH1 in HepM were significantly (2.5- to 4.5-fold) lower than those of GDH1 in RLM (Table 3).

**Table 3:** Kinetic parameters of GDH with ADP derived from the MWC equation.

**GDH kinetic parameters**	**pH 7.2**		**pH 7.5**	
	**HepM**	**RLM**	**HepM**	**RLM**
*V*_max_, mU/mg protein	203 ± 65^*^ (3)	878 ± 86 (5)	182 ± 51^*^ (5)	750 ± 151 (4)
*K*_m_ ${\text{NH}}_{4}^{+}$, mM	11.2 ± 3.5 (3)	18.0 ± 11 (5)	9.3 ± 7.2 (4)	8.8 ± 4.1 (4)
*V*_max_/*K*_m_ ${\text{NH}}_{4}^{+}$, min^−1^ mg^−1^ mL^−1^	0.018	0.048	0.019	0.085
*n*	2.6 ± 0.3 (3)	3.8 ± 1.0 (5)	2.9 ± 0.7 (4)	2.7 ± 0.3 (4)
*L_0_*	3,387 ± 4,165 (3)	5,120 ± 9,017 (5)	4,705 ± 3,876 (4)	207,232 ± 378,128 (4)
*K*_a_ ADP, mM	0.42 ± 0.26 (3)	0.51 ± 0.14 (5)	0.44 ± 0.37 (3)	0.11 ± 0.14 (4)

*Statistical analysis was performed using Student t-test*.

* *P < 0.001 vs. RLM. The number of independent preparations assayed is indicated between parentheses*.

**Figure 7:**
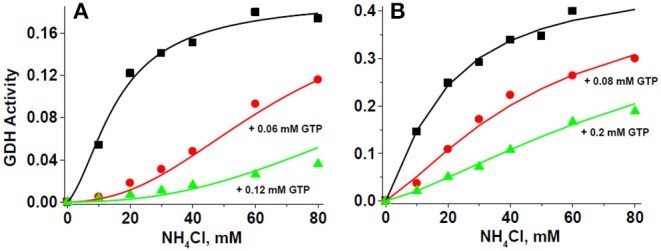
GTP inhibition of GDH activity fits the MWC equation. For these representative experiments, 279 μg protein of HepM **(A)** and 86 μg protein of RLM **(B)** were incubated in KME + HEPES buffer at pH 7.50 and 37°C. The solid lines represent the simultaneous global fitting of all experimental points to the exclusive ligand binding MWC Equation 1. The χ^2^ values of the non-linear regression analyses were 0.00005 and 0.00013 for HepM GDH and RLM GDH, respectively. The *V*_max_ values in ΔAbs/min at saturating ADP concentration were 0.199 ± 0.013 (1.5 mM ADP) for HepM GDH; and 0.471 ± 0.041 (2.1 mM ADP) for RLM GDH.

Data of the GTP inhibition on GDH1 activity also fitted well to the MWC Equation 1 (Figure 7). These experiments were carried out in the presence of saturating ADP. This was the reason why *L* values were now too low (Table 4); that is, ADP transformed most of the inactive T enzyme forms into active R forms. Nevertheless, GTP was still able to exert a potent inhibitory effect on the reverse GDH1 activity, with *K*_i_ values in the low micromolar range. It is noted that the *K*_m_ values for ammonium derived from the GTP allosteric inhibition (Table 4) were highly similar to those derived from the ADP allosteric activation (Table 3), which provided further validation to the MWC Equation 1 that it can accurately reproduce the GDH1 kinetic behavior.

**Table 4:** Kinetic parameters of the GDH inhibition by GTP derived from the MWC equation.

**GDH kinetic parameters**	**pH 7.2**		**pH 7.5**	
	**HepM**	**RLM**	**HepM**	**RLM**
*V*_max_, mU/mg protein	163 ± 78^*^ (3)	848 ± 119 (3)	178 ± 56 (3)	714 (2)
*K*_m_ ${\text{NH}}_{4}^{+}$, mM	12.0 ± 2.6 (3)	8.5 ± 4.5 (3)	10.3 ± 2.6 (3)	5.6 ± 3.6 (3)
*n*	1.99 ± 0.65 (3)	2.17 ± 0.25 (3)	3.3 ± 1.3 (3)	1.6 ± 0.2 (3)
*L'*	8.8 ± 11 (3)	20 ± 28 (3)	31 ± 34 (3)	14 ± 20 (3)
*K*_i_ GTP, μM	28 ± 27 (3)	60 ± 10 (3)	64 ± 59 (3)	31.7 ± 7 (3)

* *P < 0.001 vs. RLM. The number of independent preparations assayed is indicated between parentheses*.

### Assessment of the GDH Reverse Reaction Activity *in vivo*

For estimation of the GDH1 activity under physiological conditions, Equation 2 was applied. This equation represents the concerted transition model of MWC for exclusive ligand binding including GTP inhibition and ADP activation (Equation 1), together with ordered Bi-Bi Michaelis–Menten terms for 2-OG and NADPH. *L* is the allosteric transition constant; *K*_aADP_ is the activation constant for ADP, and *K*_iGTP_ is the inhibition constant for GTP.

(2) $$\begin{array}{l} v=\text{V max}\left(\frac{\frac{\left[\text{NADPH}\right]}{{Km}_{\text{NADPH}}}\frac{\left[2\text{OG}\right]}{{Km}_{2\text{OG}}}}{1+\frac{\left[\text{NADPH}\right]}{{Km}_{\text{NADPH}}}+\frac{\left[\text{NADPH}\right]}{{Km}_{\text{NADPH}}}\frac{\left[2\text{OG}\right]}{{Km}_{2\text{OG}}}}\right)\times \\ \;\left(\frac{\frac{\left[{\text{NH}}_{4}^{+}\right]}{{Km}_{\text{NH}4+}}{\left(1+\frac{\left[{\text{NH}}_{4}^{+}\right]}{{Km}_{\text{NH}4+}}\right)}^{\text{n}-1}}{\frac{L{\left(1+\frac{\left[\text{GTP}\right]}{{Ki}_{\text{GTP}}}\right)}^{\text{n}}}{{\left(1+\frac{\left[\text{ADP}\right]}{{Ka}_{\text{ADP}}}\right)}^{\text{n}}}+{\left(1+\frac{\left[{\text{NH}}_{4}^{+}\right]}{{Km}_{\text{NH}4+}}\right)}^{\text{n}}}\right) \end{array}$$

The metabolite concentrations either determined in the present study or reported (Table 5) as well as the kinetic parameters here determined (Tables 2–4), together with Equation 2, were used to predict the GDH1 activity under *in vivo* conditions. It should be noted that the ATP content determined in HepM and RLM (Table 5) was attained in mitochondria incubated in the absence of external nucleotides, which is not a physiological condition. The ${\text{NH}}_{4}^{{+}_{,}}$ ATP, glutamate, and ADP contents in RLM were similar to other previously reported (46–48).

**Table 5:** Contents of intramitochondrial metabolites.

		**HepM**		**RLM**	
**Metabolites (mM)**		**+ Gln + Pyr-Mal**	**+ Pyr-Mal**	**+ Gln + Pyr-Mal**	**+ Pyr-Mal**
2-Oxoglutarate		2.1 (2)	0.5 (1)	N.M. 0.4–1^6, *a*^	N.M. 0.11^1, d^
${\text{NH}}_{4}^{+}$ in		1.1 ± 0.5 (3)	<0.1 mM (3)	2.4 ± 2.3 (3) 5^4, a^	0.9 ± 1 (3)
${\text{NH}}_{4}^{+}$ out		3.6 ± 1.1 (3)	0.1 ± 0.2 (3)	0.9 ± 0.7 (3) 0.4 ^4, a^	0.1 ± 0.1 (3)
ATP		0.6 ± 0.1 (4)	0.7 ± 0.01 (3)	2.3 ± 0.2 (3) 10^5, b^ 7.7–9^6, *a*^	1.5 ± 0.4 (3) 6.3^3, c^
ADP	Without external ADP	0.6 ± 02 (4)	0.6 (1)	0.7 (2)	N.M.
	With external ADP	0.7 (2)	N.M.	1.7 (2) 5.3 ^5, b^ 7.3–8.9^6, *a*^	N.M.
GTP		0.14 (2)	N.M	N.M.	N.M. 0.15–0.2^3, c^
Glutamate		9.5 (2)	2.6 (2)	1.9 (1) 10–11^6, *a*^	1.6 (1)
NAPDH		N.M.	N.M.	N.M. 1.2^2.a^ 4.8–4.9^6, *a*^	N.M.
NADP^+^		N.M.	N.M.	N.M. 0.5^2, a^ 0.1–0.35^6, *a*^	N.M.

*Mitochondria were incubated for 10 min as described in Materials and Methods with either 4 mM glutamine, 1 mM pyruvate, and 2 mM malate (Gln+Pyr-Mal) or 1 mM pyruvate and 2 mM malate (+Pyr-Mal). When mitochondria were exposed to external ADP, 5 mM ADP was added after 10 min incubation; 2 min later, the reaction was stopped, and intramitochondrial ADP was determined. See Materials and Methods for a detailed description of the procedure used for the preparation of mitochondrial extracts and determination of metabolites. N.M., not measured. The number of independent preparations assayed is indicated between parentheses. Values taken from ^1^Moreno-Sánchez et al. (27), ^2^Liu and Kehrer (49), ^3^Smith et al. (50), ^4^Wanders et al. (47), ^5^Akerboom et al. (48), ^6^Wanders et al. (46). Amino acids used in the published data were ^a^glutamate (2–20 mM) or ^b^alanine (10 mM) plus malate (2 mM). In the absence of amino acids, the substrates were ^c^2-OG (1 mM) plus malate (1 mM) or ^d^pyruvate (1 mM) plus malate (2 mM)*.

**Figure 8:**
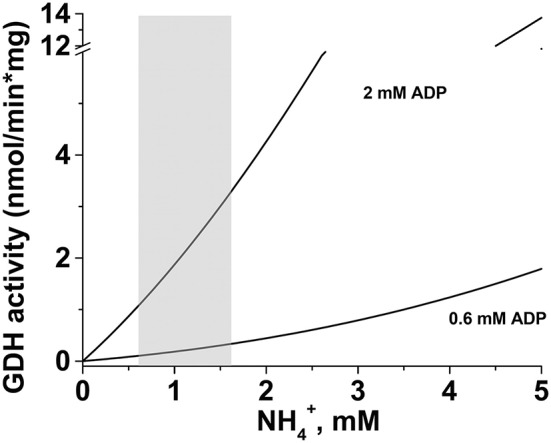
Simulation of GDH activity in the presence of modulators. For estimation of the GDH activity under physiological conditions, Equation 2 was used. The kinetic parameters at pH 7.5 were taken from Tables 2, 3, and concentrations of substrates and modulators were taken from Table 4. The gray-shaded area represents the range of ${\text{NH}}_{4}^{+}$ concentrations that can be found in cancer mitochondria when glutamine is oxidized (Table 4). *In silico* analysis was performed in the OriginPro 8 software with the following concentration values: 2.1 mM 2-oxoglutarate, 0.6 or 2 mM ADP, 0.14 mM GTP, and 1.2 mM NADPH.

Of greater significance for the present study, the ADP content was lower in HepM than in RLM (Table 5). Then, at 0.6–1.6 mM ${\text{NH}}_{4}^{+}$ and in presence of 0.6 mM ADP, the mitochondrial GDH activity in the aminating reverse reaction would be of 0.11 to 0.34 nmol/min ^*^ mg protein, whereas in presence of 2 mM ADP the activity would range from 1.1 to 3.3 nmol/min ^*^ mg protein in HepM (Figure 8).

## Discussion

### Ammonium Promotes Metastatic Cancer Cell Proliferation

Ammonium supplementation to glutamine-containing culture media was able to stimulate growth of the highly malignant HeLa, MDA-MB-231, PC3, HCT116, and Colo205 cancer cells in 2-D cultures. These human cancer cell lines are metastatic and hence have heightened abilities for migration, invasion, and colonization in glutamine-containing media (51–56). They also show enhanced drug resistance due to overexpression of plasma membrane multidrug pumping ATPases, including P-glycoprotein and multidrug resistance protein-1 (57). Interestingly, MCF-7 cells, a human breast cancer cell line with an attenuated metastatic potential (51), as well as SiHa and A549 cells, showed no significant growth stimulation by ammonium addition to glutamine-containing media. In a previous study, it was also reported that ammonium (2–5 mM) only slightly stimulated MCF-7 cell proliferation (21).

A less clear effect of ammonium on 3-D cancer cell growth was probably due to the development of hypoxic and hypoglycemic areas within MCTS (58); greater hypoxic areas develop in greater MCTS such as those formed by HeLa cells. Prolonged hypoxia of cancer cells induces severe suppression of mitochondrial functions (59). Because ammonium assimilation is primarily a mitochondrial function, and GDH1 and a glutamine synthetase isoform (another enzyme able to incorporate ammonium) are localized in the mitochondrial matrix, the putative ammonium assimilation in cancer cells could be impaired when the mitochondrial function is compromised.

Metastatic cancer cells were best equipped for ammonium assimilation than non-metastatic cancer cells in glutamine-containing culture media. This interesting observation was not further examined in the present study, but it might reflect an essential requirement during migration, invasion, and/or colonization. Indeed, GDH and glutamine synthetase have been proposed as sensitive markers of metastasis in colorectal and ovarian cancers, respectively (23, 25). This issue clearly deserves further experimental analysis.

### GDH Covalent Modification–Activity Relationship

It was previously demonstrated by using short hairpin RNA technology and performing metabolomic tracing analysis of [^15^N]amide-glutamine metabolism that GDH was the primary step of ammonium assimilation in human breast cancer MCF-7 and T47D cells (5). Increased tumor spheroid growth prompted by ammonium was suppressed by GDH down-regulation; the alternative ammonium assimilation routes catalyzed by carbamoyl phosphate synthetase I and glutamine synthetase were unable to rescue the increased spheroid growth with ammonium.

It has been reported that gene expression of GDH1 and glutaminase is increased by ammonium in the culture medium of metastatic Hep3B (human hepatoma) cells under normoxia (20). Moreover, the levels of GDH mRNA and protein are significantly higher in metastatic cancers vs. low metastatic cancers (19, 23, 26). GDH gene transcription was not assessed here. Likewise, ammonium supplementation significantly increased the GDH protein levels in the metastatic cancer HeLa, DU145, and MDA-MB-231cells, whereas it did not change in the low metastatic MCF-7 and metastatic Colo 205 cells. In addition, the degree of GDH (Ser/Thr) phosphorylation and (Lys) acetylation did markedly increase in HeLa and MDA-MB-231cells. All these changes in metastatic cells enhance GDH activity.

Phosphorylation of GDH1 seems to confer greater catalytic efficiency (*V*_max_*/K*_m_, increased *V*_max_ and lower *K*_m_ for NADP^+^) and structural stability (38). However, in this last study, protein phosphorylation was not directly assessed, but it was rather assumed, and the changes determined in *V*_max_ and *K*_m_ were marginal. Ammonium supplementation certainly induced increased GDH covalent modification in 2-D HeLa and MDA-MB-231 cells. In addition, Ser phosphorylation was the covalent modification that better correlated with GDH activity, because it was approximately three times lower in HepM vs. RLM (Figure 4), which was similar to the difference in activity (Tables 2–4). Furthermore, direct GDH phosphorylation did slightly affect *V*_max_, indicating that covalent modifications might regulate GDH activity. Perhaps, phosphorylation and acetylation of GDH might also regulate its stability or subcellular localization, but this issue clearly requires further experimental analysis.

### Kinetic Properties of Tumor Mitochondria GDH1

To have an accurate estimate of the level of active enzyme under *in vivo* conditions, with the physiological covalent modifications included, *V*_max_ must be determined in intact cells and mitochondria rather than in isolated enzymes to revealing the actual content of active enzyme expressed. AS-30D hepatoma cells are a cancer cell model that allows for preparing functional, tightly coupled mitochondria with high yields; this cannot be achieved with human cancer cell cultures using the available commercial kits for preparing mitochondria. Thus, mitochondrial matrix enzymes from cancer cells can be readily and reliably analyzed. A systematic kinetic analysis of GDH1 activity in hepatoma mitochondria was undertaken to elucidate its kinetic parameters, including *K*_m_ values for ${\text{NH}}_{4}^{+}$, 2-OG, and NADPH; catalytic efficiencies (*V*_max_/*K*_m_); *K*_a_ values for ADP and leucine; *K*_i_ value for GTP; Hill coefficient *n*; and allosteric transition constant *L*. For comparison, liver mitochondria GDH1 was also characterized.

The *V*_max_ values for the reverse reaction were near one order of magnitude greater than those determined for the forward reaction with NADP^+^ (27). Higher *V*_max_ values for the GDH1 reverse reaction than for the forward reaction have been also previously described by others (4, 9). Thus, at least from a kinetic standpoint, GDH can readily be able to catalyze the assimilatory reaction with ammonium under near-physiological conditions.

Many mutations described for kidney GDH lead to a diminished ability of GTP to affect activity (3). However, HepM GDH1 was equally sensitive to GTP inhibition than RLM GDH1 indicating that both enzymes showed similar GTP *K*_i_ values. Moreover, other ligand binding constants (*K*_m_, *K*_0.5_, *K*_a_) were also similar between both enzymes. The high similarity of the GDH binding parameters between HepM and RLM clearly indicated that the GDH1 isoform expressed in hepatoma cells has no mutations, or if mutations occurred, they were silent as no functional consequences were apparent. The only difference was the lower content of GDH1 active protein in HepM; that is, its *V*_max_ and catalytic efficiency (*V*_max_/*K*_m_) values were clearly lower, which derived from both covalent modifications and lower protein content.

In several studies where GDH activity has been determined, ADP has been used at 1 mM. However, it should be noted that such ADP concentration is not saturating for GDH at pH of 7–7.5, and hence greater concentrations are required (>2 mM) to take the enzyme to its fully active R form. On the other hand, the pH profile of the GDH1 reverse activity suggested the involvement of histidine and cysteine residues in GDH catalysis. From previous studies (60–63), it has been indicated that indeed His and Cys, as well as Lys residues, are involved in GDH catalysis (deamination/amination) and 2OG/Glu binding. However, in these last studies, ADP was used at non-saturating concentrations when assessing activity, and hence, full display of kinetic properties was not achieved. Further inactivation analysis, pH profile, and site-directed mutagenesis studies should help in clarifying the nature of the actual residues involved in both catalysis and ligand binding.

The purified bovine GDH has six subunits, each with binding sites for substrates, and pyridine and purine nucleotides. Subunit cooperative interaction is promoted by ADP and inhibited by GTP. The Hill coefficient (*n*_*H*_) values, determined to be 2.3–2.8 when varying ADP (Table 2), suggested that GDH has a moderate cooperativity among its six subunits (32, 35). In turn, the GDH kinetics fitted to the concerted MWC exclusive binding Equation 1 yielded *n* values near 3 when varying ${\text{NH}}_{4}^{+}$ and ADP (Table 3). Then, according to the MWC model, in the absence of essential activators, the six enzyme subunits were in the inactive, tense (T) state, whereas, in the presence of ADP, the enzyme progressively transforms up to three subunits into the active, relaxed R state. Therefore, as GDH is structured as an association of two trimers, an explanation of the kinetic data might be that the enzyme behaved as a dimer of two independent or semi-independent trimers.

### GDH Reverse Reaction Under Physiological Conditions

NAD^+^ and NADP^+^ have similar standard redox potentials, but NAD^+^ predominantly serves for catabolism and ATP generation, whereas NADPH is the main reducing agent for biosynthetic pathways (64). The mammalian liver and brain GDHs show similar affinities for NAD^+^ (*K*_m_ = 0.17–0.83 mM) and NADP^+^ (*K*_m_ = 0.16–1.22 mM), as well as for NADH (*K*_m_ = 0.028–0.12 mM) and NADPH (*K*_m_ = 0.022–0.12 mM) (32, 33, 44, 45, 65, 66). In consequence, both NADH and NADPH are possible products of the forward GDH reaction, as well as substrates of the reverse reaction. The high activity and high affinity for NADH of the respiratory chain (67) do not allow the mitochondrial matrix NADH content to build up, and this is the reason why the NADH/NAD^+^ ratios are low (0.05–0.18) in functional mitochondria (31, 46, 49, 68). In turn, NADH cannot be used by the enzymes involved in the GSH/oxidative stress metabolism, whereas NADPH is not a respiratory chain substrate. Then, the GDH reverse reaction with NADH as cosubstrate is unfavorable. In contrast, the intramitochondrial NADPH/NADP^+^ ratios are usually around one or even higher (46, 49). Thus, the GDH reverse reaction with NADPH is more likely to occur than with NADH under physiological conditions. Under prolonged hypoxia, oxidative phosphorylation is depressed, and NADH and ADP accumulate, further favoring the GDH reverse reaction.

There are also reports (22, 69–71) stating that GDH preferentially catalyzes the forward, oxidative deamination reaction than the reverse, reductive amination reaction in cancer cells. However, the actual *K*_EQ_ value of the GDH reaction indicates that the reverse reaction is thermodynamically favored when at least micromolar ammonium concentrations are present. Therefore, with millimolar ammonium concentrations within cancer cells (Ehrlich ascites mouse cells, 0.4–2.3 mM) (72) and in the surrounding microenvironment (0.8–3 mM) (5), the only possible GDH reaction, regardless of the GDH isoform and subcellular localization, is that of N assimilation.

The data of the present study reveal that the kinetic properties of GDH1 make feasible the reverse reaction under physiological conditions. For instance, the GDH reverse reaction is favored by its higher *V*_max_ value (vs. forward reaction *V*_max_); that is, GDH1 has a higher catalytic capacity for its reverse reaction. The reverse GDH reaction is also thermodynamically favored in the presence of micromolar ammonium concentrations and physiological mitochondrial matrix Glu/2-OG (1–10) and NADPH/NADP^+^ (~1) ratios, despite its high *K*_m_ values for ammonium, which were above the physiological range of concentrations. In this last regard, it was here determined that cancer mitochondria actively produce ammonium from glutamine, in a reaction catalyzed by glutaminase, leading to higher mitochondrial matrix and extramitochondrial ammonium concentrations, which in turn are sufficient to drive the GDH reverse reaction. An alternative and supplementary source of ammonium might be AMP deamination in the cytosol, which is also enhanced in cancer cells (73). Thus, the emerging scenario for glutamate/glutamine metabolism in cancer mitochondria seems to privilege the conservation and further formation of glutamate as an N-carrier/donor, in which both glutamine and 2-OG are transformed into glutamate.

Interestingly, treatment with the so-called ammonia-scavenging drugs (phenylacetate and phenylbutyrate, which in fact conjugate with glutamine to form phenylacetylglutamine and phenylbutyrylglutamine, which are excreted) arrests growth of prostate cancer, renal cancer, and leukemia cell lines (74, 75). Phenylbutyrate also decreases by 35 to 45% the size of tumors in rodents (76). However, phenylacetate has shown a negligible antitumor activity in clinical trials (77).

Total GDH activity in intact cancer cells was approximately one-third of the GDH1 activity determined in isolated hepatoma mitochondria. Likewise, it has also been estimated that the mitochondrial volume within a cell amounts up to ~30% of total cellular volume. In consequence, the GDH activity determined in cells seemed to mostly correspond to that of the mitochondrial GDH1 isoform.

The *in silico* simulation of GDH1 activity with physiological concentrations of substrates and modulators, and using the MWC equation for exclusive binding, predicted an activity in the range of 1.1 to 3.3 nmol/min × mg (with 2 mM ADP) in isolated mitochondria. By considering that the mitochondrial fraction corresponds to 30% of cellular protein, the GDH activity scaled up to intact cells might oscillate between 0.33 and 0.99 nmol/min ^*^ mg cell protein. This GDH activity range for the reverse reaction is well within the range of fluxes determined for biosynthesis of protein (2.5–8.8 nmol/min ^*^ mg protein), glycogen (0.25–0.55 nmol/min ^*^ mg protein), urea (2.4–28 nmol/min ^*^ mg protein), and fatty acids (0.09–10.7 nmol/min ^*^ mg protein), or it is even above those of cholesterol (0.014–0.025 nmol/min ^*^ mg protein) and nucleotides (0.1 nmol/min ^*^ mg protein), in normal and tumor cells (78–83). Then, it seems thermodynamically and kinetically feasible that GDH may contribute to accelerate cancer cell proliferation by providing glutamate, through its reverse ${\text{NH}}_{4}^{+}$ fixing reaction.

Ammonium, at millimolar concentrations, induced increased growth rates of metastatic cancer cells, but not of non-cancer cells in which it was toxic. The kinetic properties of GDH, as well as the thermodynamically favorable GDH reverse reaction, when at least micromolar ammonium concentrations are present, support that the physiological GDH role in cancer cells is to catalyze the ${\text{NH}}_{4}^{+}$ fixation to promote proliferation. The MWC equation predicted, at physiological concentrations of substrates and modulators, a rate of ammonium assimilation catalyzed by tumor GDH completely compatible with the anabolic rates required for active cell proliferation.

## Materials and Methods

### Chemicals

Ammonium chloride, ADP, DTT, EDTA, GTP, glutamate, glutamine, 2-OG, 3-phosphoglycerate, l-leucine, NAD^+^, NADP^+^, NADPH, NADH, Triton X-100, MgCl2, MOPS, HEPES, Tris, imidazole, acrylamide, GDH (no. G2626), rabbit muscle phosphorylase kinase (no. P2014), and agarose were from Sigma Chem. Co. (St. Louis, MO, USA). Anti-GDH1/2 (no. sc-160383); anti-α-tubulin (no. sc-5286); antiacetylated lysine (no. ab190479); anti-P-Ser (Q5phospho-ser; Qiagen no.37430); and anti-P-Thr (Q7phospho-Thr; Qiagen no.37420) were purchased from Santa Cruz (sc) Biotechnology (Santa Cruz, CA, USA), Abcam (ab) (Cambridge, MA, USA), or Qiagen (Venlo, the Netherlands). Glyceraldehyde-3-phosphate dehydrogenase (GAPDH, no. 105686) and HK (no. 11426362001) were from Roche (Mannheim, Germany). Recombinant phosphoglycerate kinase (PGK) was from *Entamoeba histolytica* (84).

### Cell Growth and Culture

Propagation and isolation of AS-30D ascites hepatoma cells and culture of human cervix cancer HeLa and SiHa cells, human breast cancer MDA-MB-231 and MCF-7 cells, human prostate cancer PC3 and DU145, human lung A549, human colorectal HCT116 and Colo 205 cells, human HFF-1 fibroblasts, and mouse 3T3 fibroblasts were carried out in Dulbecco modified eagle medium (DMEM) plus 25 mM glucose as previously described (51, 55, 59, 85–87). All cancer and non-cancer cell lines used were purchased from ATCC and cultured in DMEM (Sigma-Aldrich) supplemented with 10% fetal bovine serum (Biowest, Nuaillé, France) and 10,000 U penicillin/streptomycin (Sigma-Aldrich) and placed under a humidified atmosphere of 5% CO_2_/95% air at 37°C. Genotyping (INMEGEN, Tlalpan, Mexico city, Mexico) of the cancer cell lines showed >90% of the canonic allelic markers displayed in the ATCC original clones.

For 2-D cultures, 20 × 10^3^ cells/well were grown in 96-well plates in the presence or absence of different ammonium chloride concentrations; the addition of NH_4_Cl pH 7.0 did not change the pH of the culture medium (DMEM + 25 mM glucose). Cell growth was followed by counting cellular density every 24 h during 7 to 8 days. Viability was determined by the trypan blue assay, which revealed <10% cellular death (54). The duplication time was determined by using the following equation: $n=\frac{1}{\frac{\left[3.32\left(\log N_{F}-\log N_{I}\right)\right]}{\left(t_{F}-t_{I}\right)}}$, where *N*_*F*_ represents the number of cultured cells at the end of the exponential growth phase; *N*_I_ represents the number of cells at the beginning of the growth curve; *t*_F_ is the final time at which cells were harvested, and *t*_I_ is the initial culture time. The specific growth rate μ was calculated from the slope of a semilogarithmic plot of cell densities in the exponential growth phase vs. time (88).

For tridimensional (3-D) cultures, HeLa and MDA-MB-231 cells (2 × 10^4^ cells/mL) were seeded in 2% (wt/vol) agarose-coated culture dishes in 5 mL DMEM (+ 25 mM glucose) with the indicated ammonium chloride concentrations. After 5 days, the culture medium was refreshed, and the MCTSs formed were placed under slow orbital shaking (20–50 rpm) at 37°C and 95% air/5% CO_2_. Fresh culture medium with 25 mM glucose ± NH_4_Cl was replaced every 3 days, which helped to discard incompletely formed spheroids. The spheroid growth was determined at the indicated times by measuring MCTS diameter with a calibrated reticule (1/10 mm) in an inverted phase contrast microscope (Zeiss, Thornwood, NY, USA) (86).

### Isolation of Mitochondria

Tightly coupled mitochondria were isolated from fed-rat liver (RLM) and AS-30D cells (HepM) as described elsewhere (89, 90). Both mitochondrial preparations were subjected to further dilution in SHE buffer (250 mM sucrose, 10 mM HEPES, 1 mM EGTA, pH 7.3) and centrifugation (12,857 × g for 10 min at 4°C); these steps were repeated thrice to minimize the presence of contaminating cytosolic proteins. The resulting mitochondrial fractions were resuspended at 30 to 80 mg protein/mL in SHE buffer with 1 mM PMSF, 1 mM EDTA, 5 mM DTT, and 10% glycerol, and stored at −70°C until use for determination of enzyme activity and Western blotting. Animal manipulation was carried out in accordance with the recommendations stated by the Mexican Official Standard NOM-062-ZOO-1999 norm.

### GDH Activity

The GDH activity assay for the reverse reaction was determined at 37°C in KME (120 mM KCl, 20 mM K-Mops, 1 mM K-EGTA) buffer at pH 7.2, or in KME buffer + 10 mM HEPES at pH 7.5, and in the presence of 0.8 mM MgCl_2_, 0.02% Triton X-100, 0 to 2.4 mM ADP, 0–0.75 mM 2-OG, 0.15–0.2 mM NADPH, and 0.07–0.1 mg protein for RLM or 0.2–0.3 mg protein for HepM; for the saturation curves with NADPH, 25 to 30% lower protein contents were used. The specific GDH reaction was started by adding 5–100 mM NH_4_Cl. Negligible spurious consumption of NADPH was attained under the described conditions; presence of significant levels of GDH ligands derived from the mitochondrial matrix can be discarded because of the large dilution of the mitochondrial preparation in the reaction assay (at least 40 times and usually 100 times or more). The decrease in the absorbance at 340 nm was followed for several minutes (~10 min) to allow for full development of the pronounced enzyme hysteretic behavior, although the initial signal decrease (1–3 min) was taken to calculate the GDH rates. The protein concentration ranges used for each mitochondrial type were well within the linearity range of enzyme activity. To calculate the kinetic parameters, the experimental data were fitted by non-linear regression analysis to the Hill or MWC equation, using the Microcal Origin 5.0 software (OriginLab, Northampton, MA, USA).

### Western Blotting and Immunoprecipitation Assays

Mitochondria were solubilized in RIPA lysis buffer [phosphate-buffered saline 1 × pH 7.2, 1% IGEPAL NP40, 0.1% sodium dodecyl sulfate (SDS), and 0.05% sodium deoxycholate] plus 1 mM of PMSF (phenyl methanesulfonyl fluoride) and one tablet of complete protease inhibitors cocktail (Roche) and subjected to SDS–polyacrylamide gel electrophoresis in 12.5% polyacrylamide gels. The proteins were immobilized on polyvinylidene fluoride membranes and immunoblotted with human anti-GDH (1:1000 dilution); specific proteins were revealed with peroxide-conjugated secondary antibodies (anti-goat, no. sc-2768; anti-rabbit, no. sc-2317; anti-mouse, no. sc-2005), followed by chemiluminescence detection as previously described (91). Covalent GDH modification was assessed by initially immunoprecipitating with the specific anti-GDH antibody followed by detection with antibodies anti–phospho-Ser, anti–phospho-Thr, and anti-acetylLys as previously described (27).

### Determination of Metabolites

Freshly prepared mitochondria (10 mg protein/mL) were incubated in KME buffer + 2 mM K-phosphate at 37°C under smooth orbital shaking with either 1 mM pyruvate + 2 mM malate or 4 mM glutamine + 1 mM pyruvate + 2 mM malate. After 10 min, aliquots were withdrawn, mixed with ice-cold KME buffer, and centrifuged at 17,000 × g for 1 min at 4°C. The supernatant was mixed with ice-cold 3% (vol/vol) perchloric acid (PCA) in 1 mM EDTA and kept in ice. The mitochondrial pellet was resuspended in cold KME buffer and centrifuged at 17,000 × g for 1 min at 4°C. This procedure was repeated once. The final mitochondrial pellet was mixed with ice cold 3% PCA/1 mM EDTA. The two fractions were neutralized with 3 M KOH/0.1 mM Tris and stored at −72°C until use for determination of ammonium, 2OG, Glu, ADP, and ATP by standard enzymatic methods (92, 93).

The intramitochondrial GTP content was estimated from the difference between the content of ATP + GTP determined by assay with *Eh*PGK (which can take either GTP or ATP as substrate, with 0-fold higher affinity for the first) and ATP content determined by assay with HK. It was assumed that HK does not use GTP as substrate. The stock solutions of GTP, ADP, 2-OG, ${\text{NH}}_{4}^{+}$, and Glu were also routinely calibrated by standard enzymatic methods (92, 93). The GTP stock was prepared in presence of 1 mM EDTA, making it stable for several weeks. GTP in the stock solution was determined in a coupled enzymatic assay with *Eh*PGK (2 U) and GAPDH (2 U) in presence of 5 mM MgCl2, 1 mM EDTA, 2 mM DTT, 0.15 mM NADH, and 2 mM 3-phosphoglycerate.

### Statistical Data Analysis

The data represent the mean ± standard deviation (SD) of at least three independent cell preparations (n). For statistics between two experimental groups, Student *t*-test analysis was used (94). For statistics between three or more experimental groups, one-way analysis of variance (ANOVA)/*post hoc* Scheffé analysis was used (94, 95). For both, *P* < 0.05 was used as statistical significance criterion.

## Data Availability Statement

All datasets generated for this study are included in the article.

## Author Contributions

RM-S and ÁM-H: conceptualization. RM-S, ÁM-H, JG-P, SP-V, DR-C, and JP-F: investigation. RM-S and ÁM-H writing—original draft preparation. RM-S, ÁM-H, ES, and SR-E: writing—review and editing.

### Conflict of Interest

The authors declare that the research was conducted in the absence of any commercial or financial relationships that could be construed as a potential conflict of interest.
